# Melatonin inhibits attention-deficit/hyperactivity disorder caused by atopic dermatitis-induced psychological stress in an NC/Nga atopic-like mouse model

**DOI:** 10.1038/s41598-018-33317-x

**Published:** 2018-10-08

**Authors:** Gunhyuk Park, Young-Suk Jung, Moon-Ki Park, Chae Ha Yang, Yong-ung Kim

**Affiliations:** 1 The K-herb Research Center, Korea Institute of Oriental Medicine, 1672 Yuseong-daero, Yuseong-gu, Daejeon, 34054 Republic of Korea; 2 College of Pharmacy, Pusan National University, Busan, Republic of Korea; 3 Department of Pharmaceutical Engineering, College of Biomedical Science, Daegu Haany University, 290 Yugok-dong, Gyeongsan-si, Gyeongsangbuk-do, 38610 Republic of Korea; 4 Department of Physiology, College of Korean Medicine, Daegu Haany University, Daegu, Republic of Korea

## Abstract

Atopic dermatitis (AD) is a chronic inflammatory skin disease with the hallmark characteristics of pruritus, psychological stress, and sleep disturbance, all possibly associated with an increased risk of attention-deficit/hyperactivity disorder (ADHD). However, the etiology of the possible association between AD and ADHD is still not well understood. 2,4-dinitrochlorobenzene or corticosterone was used to evaluate the atopic symptom and its psychologic stress in the atopic mice model. Melatonin, corticotropin-releasing hormone, corticotropin-releasing hormone receptor, urocortin, proopiomelanocortin, adrenocorticotropic hormone, corticosterone, cAMP, cAMP response element-binding protein, dopamine and noradrenaline were analyzed spectrophotometrically, and the expression of dopamine beta-hydroxylase and tyrosine hydroxylase were measured by Western blotting or immunohistochemistry. AD-related psychological stress caused an increase in the levels of dopamine beta-hydroxylase and tyrosine hydroxylase, degradation of melatonin, hyper-activity of the hypothalamic-pituitary-adrenal axis, and dysregulation of dopamine and noradrenaline levels (ADHD phenomena) in the locus coeruleus, prefrontal cortex, and striatum of the AD mouse brain. Notably, melatonin administration inhibited the development of ADHD phenomena and their-related response in the mouse model. This study demonstrated that AD-related psychological stress increased catecholamine dysfunction and accelerated the development of psychiatric comorbidities, such as ADHD.

## Introduction

ADHD is a common childhood disorder affecting around 7% of all children, and often persists into adulthood^1,2^. According to the Diagnostic and Statistical Manual of Mental Disorders, ADHD includes 3 subtypes: inattentive, hyperactive-impulsive, and combined. To define these subtypes, 2 symptom dimensions have been used: inattention and hyperactivity-impulsivity^3,4^. These symptoms appear with varying severity and can lead to adverse consequences, including anxiety, depression, delinquency, sleep disorders, and circadian rhythm defects^2,3,5^. Despite these consequences, the complex molecular mechanisms underlying ADHD are still understudied and unclear. The main hypothesis, centered on monoamine neurotransmission, supposes that in ADHD, complex interactions between the dopamine, noradrenaline, and serotonin systems are deregulated; human studies have associated ADHD with genes encoding monoamine receptors and transporters^6,7^. Most treatments available today include the administration of psycho stimulants that increase levels of available dopamine and are believed to restore monoaminergic balance, which is altered during ADHD development^8–10^.

Atopic dermatitis (AD) is a type of chronic inflammation of the skin characterized by eczematous skin lesions, papules, severe pruritus, and excoriations^11,12^. AD affects 10–20% of children worldwide and persists into adulthood in a minority of cases (approx. 2–3% of adults), with an increased prevalence in urbanized societies^13^. Importantly, AD is associated with other non-allergic diseases, including psychiatric and behavioral disorders^14–17^. The prevalence of depression, stress-related disorders, and anxiety are significantly higher in the AD population^18,19^. Recent data indicate that chronic AD might directly or indirectly lead to an increased risk of psychiatric comorbidities, such as ADHD^20^. Epidemiological data suggests that the global prevalence of AD and ADHD has risen parallelly, and several cross-sectional studies have indicated co-occurrence^20–22^. Furthermore, recent cohort studies have reported a temporal association between AD and ADHD development^20,22^. Children with AD have a 1.5-fold higher risk for ADHD, and the ascribed population risk for ADHD explained by AD is roughly 9%^15,20^. According to many clinicians, it is possible that the psycho-endocrine and psycho-neuroimmunological effects of AD are caused by elevated inflammatory cytokine levels, continuous sensory stimuli, disturbed sleep, and increased stress^15,20^. Currently, the etiology of the association between AD and ADHD is still not well understood.

Many studies have focused on the role of stress as a relevant trigger of AD symptoms. Patients with AD demonstrate blunted hypothalamic-pituitary-adrenal (HPA) axis responsiveness and over-reactivity of the sympathetic-adrenal-medullary system to psychosocial stress^23,24^. Considering immune-regulatory roles of the HPA axis and the sympathetic-adrenal-medullary systems, especially under stress, an aberrant responsiveness of these systems may increase susceptibility to allergic inflammation and may be a psycho-biological mechanism of stress-related aggravation of AD^24,25^. These ideas are supported by a study showing raised glucocorticoid and adrenocorticotropic hormone (ACTH) levels under stress in adults with AD, which indicate a dysfunctional HPA axis. Interestingly, response dysfunction of the HPA axis during stress exposure is accompanied by atopy-related immunological changes in response to stressors such as increased immunoglobulin E levels, altered IL4 and interferons concentrations, and aggravation of AD symptomatology^25–27^. Additionally, recent papers report that continuous high levels of corticotropin-releasing factor-related to psychological stress can trigger AD-like skin lesions^25,28,29^. Surprisingly, according to Corminas *et al*., the cortisol response to stress in adults with ADHD was not significantly different from that in the healthy controls^3^. Nevertheless, clear differences can be observed between the combined and inattentive subtypes^3,30^. Compared with the mainly inattentive adults, patients with combined ADHD exhibited a blunted cortisol response to stress^3^. In contrast, the inattentive ADHD subtype was characterized by a trend towards a higher cortisol response than that observed in the controls^3,30,31^. These reports suggest the significance of distinguishing the inattentive and combined subtypes in the diagnosis of ADHD. Further, these findings implicate dysregulation of the HPA-axis, which is involved in several physiological systems and is central to stress regulation, in the development of ADHD.

The action of glucocorticoids on many physiological systems is to mediate stress-responses, but their long-term chronic elevation impairs catecholamine systems^32–35^. Specifically, ACTH and the sympathetic nervous system stimulate the synthesis of adrenaline precursors by enhancing the activity of tyrosine hydroxylase (TH) and dopamine beta-hydroxylase (DβH), two enzymes involved in catecholamine synthesis^36–38^. ACTH also stimulates the adrenal cortex to release corticosterone (CORT), which enhances adrenaline synthesis^38,39^. Moreover, patients with AD show elevated norepinephrine concentrations, similar to the results observed for patients with ADHD^40^. Thus, several clinical studies have found associations between AD, ADHD, and attention deficits.

Thus, we hypothesized that psychological stress caused by AD can increase DβH levels, which increases overall dysregulation of dopamine and noradrenaline, creating a state of chronic psychiatric comorbidities, such as ADHD. Our previous studies indicated that atopic stress significantly accelerates neuronal injury and increases neuro-inflammation, primarily via exacerbating a chronic state of blunted glucocorticoid activity and suppressing melatonin-mediated feedback^25^. In the present study, we employed an AD model to test the aforementioned hypothesis, which included analyzing dopamine and noradrenaline imbalance.

## Materials and Methods

### Chemical

Chemical analysis was performed as previously described^25^. Additionally, rabbit and goat anti-TH and rabbit anti-DβH antibodies were purchased from EMD Millipore (Billerica, MA, USA). A mouse DβH enzyme-linked immunosorbent assay (ELISA) kit was purchased from Aviva Systems Biology (San Diego, CA). A norepinephrine and dopamine 2-CAT ELISA Kit were purchased from Rocky Mountain Diagnostic, Inc. (Colorado Springs, CO, USA). All other reagents used were of guaranteed or analytical grade.

### *In vitro* and *in vivo* models

**Figure 1:**
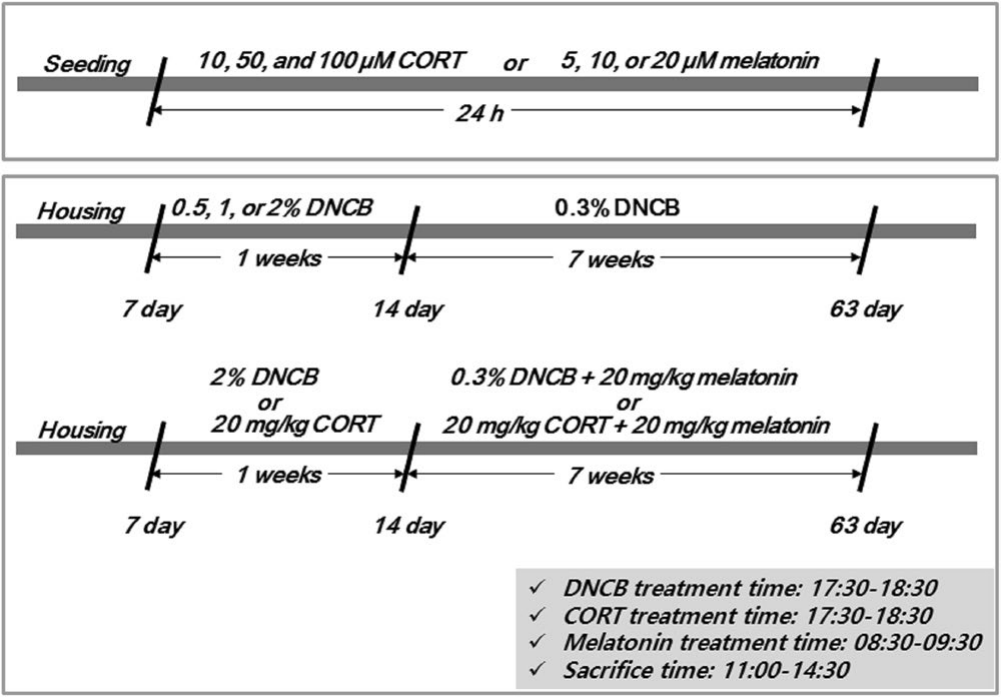
Summary of the experimental design.

Immortalized human SH-SY5Y cell culture and the 2,4-dinitrochlorobenzene (DNCB) or CORT treatment animal models were established according to previously published methods^25,41,42^. The institutional animal care committee of the Korea Institute of Oriental Medicine (KIOM) approved the experimental protocols KIOM-16-015 and KIOM-16-112. The experiments were performed according to the guidelines of the Animal Care and Use Committee at KIOM^25^. The animals were sacrificed between 11:00 a.m. and 14:30 p.m., 7 weeks after sensitization with DNCB or CORT (Fig. 1 shows the study timeline).

### Brain tissue preparation and immunofluorescence analysis

Brain tissue preparation was performed as previously described^25^. Briefly, the mice were decapitated; the skull was then removed and the brain was dissected around selected regions including the locus coeruleus (LC), prefrontal cortex (PC), and striatum (ST) using a brain matrix for kit-based analyses. All tissues were frozen *in situ* by immediate direct immersion in liquid nitrogen in order to prevent decomposition. Immunofluorescence analysis was then performed as described in a previous study^25^.

### Measurement of stress-related factors

Melatonin, urocortin (UCN), DβH, proopiomelanocortin (POMC), ACTH, CORT, corticotropin-releasing hormone (CRH), corticotropin-releasing hormone receptor 1 (CRHR1), cyclic adenosine monophosphate (cAMP), CREB phosphorylation (pCREB), norepinephrine, and dopamine were quantified using commercially available kits according to the instruction manuals or previously published methods^25^.

### Statistical analysis

All statistical parameters were calculated using Graphpad Prism 5.0 software (Graphpad Software, San Diego, CA, USA). Values are expressed as means ± standard error of the mean (S.E.M.). Statistical comparisons between the different treatments were performed using a one-way ANOVA with Tukey’s multiple comparison post hoc test. p values of <0.05 were considered to be statistically significant.

## Results

### Effects of CORT and melatonin on DβH levels in SH-SY5Y cells

**Figure 2:**
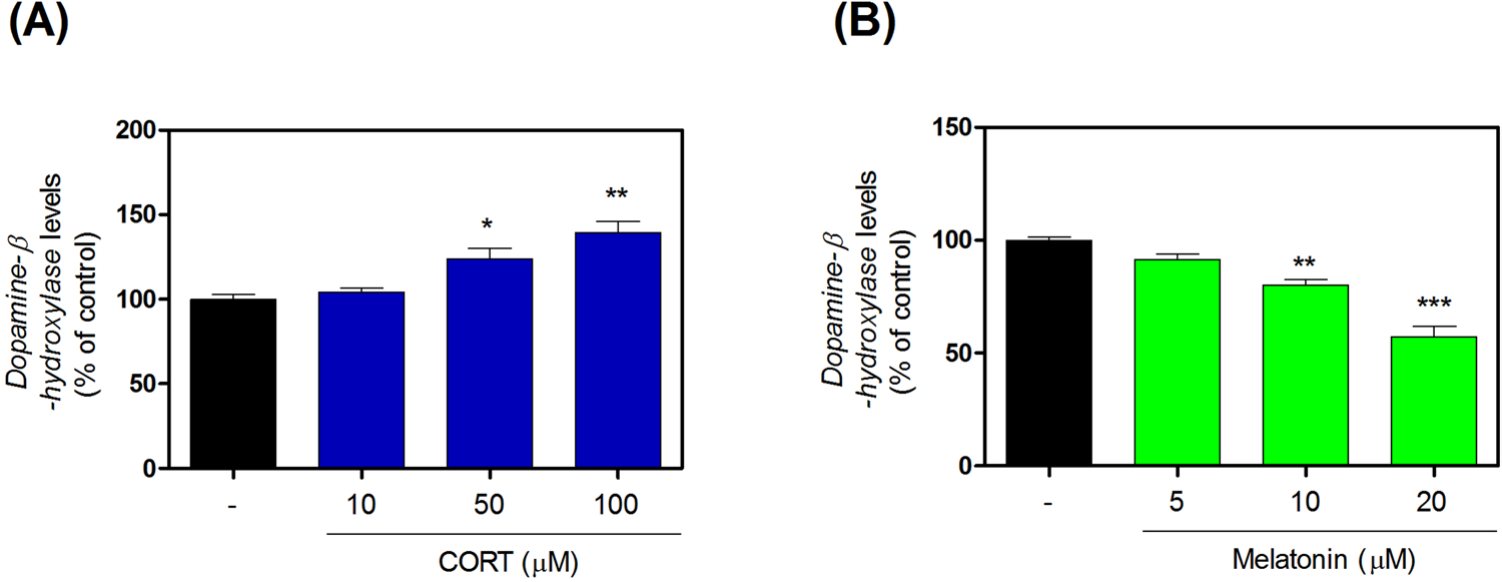
Effects of CORT on expression of DβH in SH-SY5Y cells. The levels of DβH were quantified using ELISA kits (**A**). Effects of melatonin on expression of DβH in SH-SY5Y cells. The levels of DβH were quantified using ELISA kits (**B**). Values are shown as means ± standard error of the mean. *P < 0.05, **P < 0.01, and ***P < 0.001 compared with the control group. CORT, corticosterone; DβH, dopamine beta-hydroxylase.

We measured DβH levels using ELISA kits after exposing SH-SY5Y cells to CORT or melatonin. CORT exposure significantly increased DβH levels (Fig. 2A and Supplementary Table 1) and melatonin exposure significantly decreased DβH levels (Fig. 2B and Supplementary Table 1).

### Effects of DNCB on melatonin in the LC, PC, and ST

**Figure 3:**
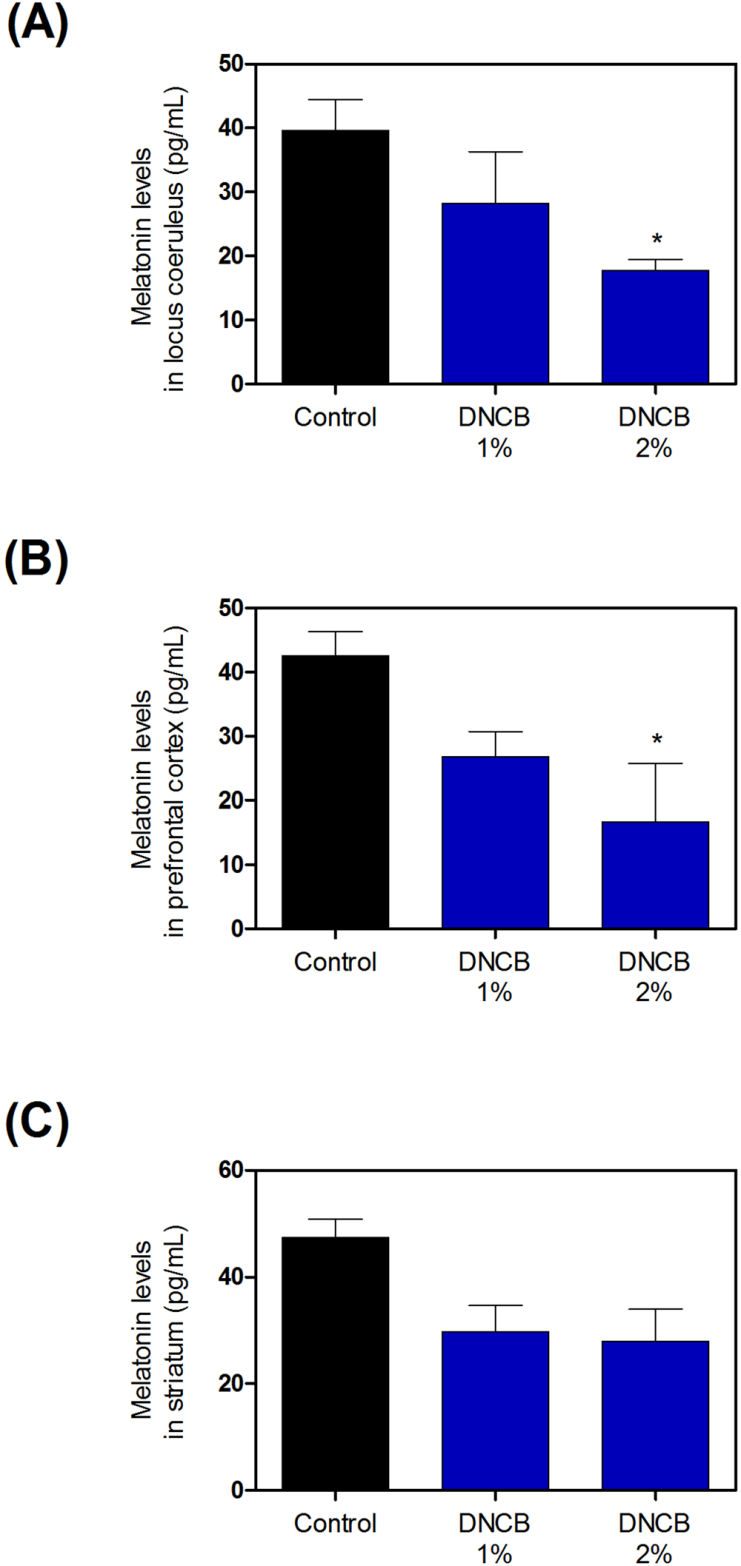
Effects of DNCB on expression of melatonin in brain. The levels of melatonin in locus coeruleus (**A**), prefrontal cortex (**B**), and striatum (**C**) of were quantified using ELISA kits. Values are presented as means ± standard error of the mean. *P < 0.05 compared with the control group. DNCB, dinitrochlorobenzene; ELISA, enzyme-linked immunosorbent assay.

We measured melatonin levels using an ELISA kit. The 1–2% DNCB groups demonstrated significantly decreased melatonin levels in the LC (Fig. 3A and Supplementary Table 2) and PC (Fig. 3B and Supplementary Table 2). However, they showed only weak decreases in the ST (Fig. 3C and Supplementary Table 2).

### Effects of melatonin on DNCB-induced HPA axis activity biomarkers

**Figure 4:**
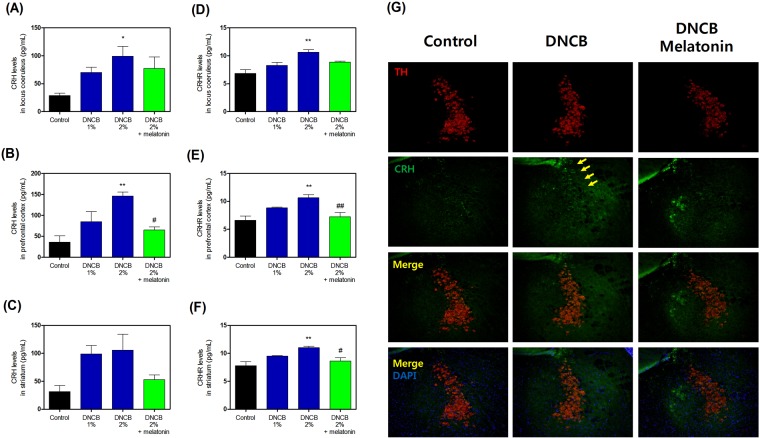
Effects of melatonin on DNCB-induced expression of CRH and CRHR in locus coeruleus, prefrontal cortex, and striatum of the brain. The levels of CRH (**A**–**C**) and CRHR (**D**–**F**) in of were quantified using ELISA kits. Representative photomicrographs are shown (**G**). Values are shown as means ± standard error of the mean. *P < 0.05 and **P < 0.01 compared with the control group; ^#^P < 0.05 and ^##^P < 0.01 compared with the 2% DNCB-alone group. DNCB, dinitrochlorobenzene; CRH, corticotropin releasing hormone; CRHR, corticotropin releasing hormone receptor; ELISA, enzyme-linked immunosorbent assay.

To determine whether melatonin affected stress hormone responses, we measured CRH and CRHR levels. Treatment with DNCB significantly increased CRH levels (Fig. 4A–C, and Supplementary Table 3), while treatment with 20 mg/kg melatonin reduced the DNCB-induced CRH level increase (Fig. 4A–C, and Supplementary Table 3). Also, treatment with DNCB significantly increased CRHR levels (Fig. 3D–F, and Supplementary Table 3), while treatment with 20 mg/kg melatonin reduced the DNCB-induced CRHR increase (Fig. 4D–F, and Supplementary Table 3).

**Figure 5:**
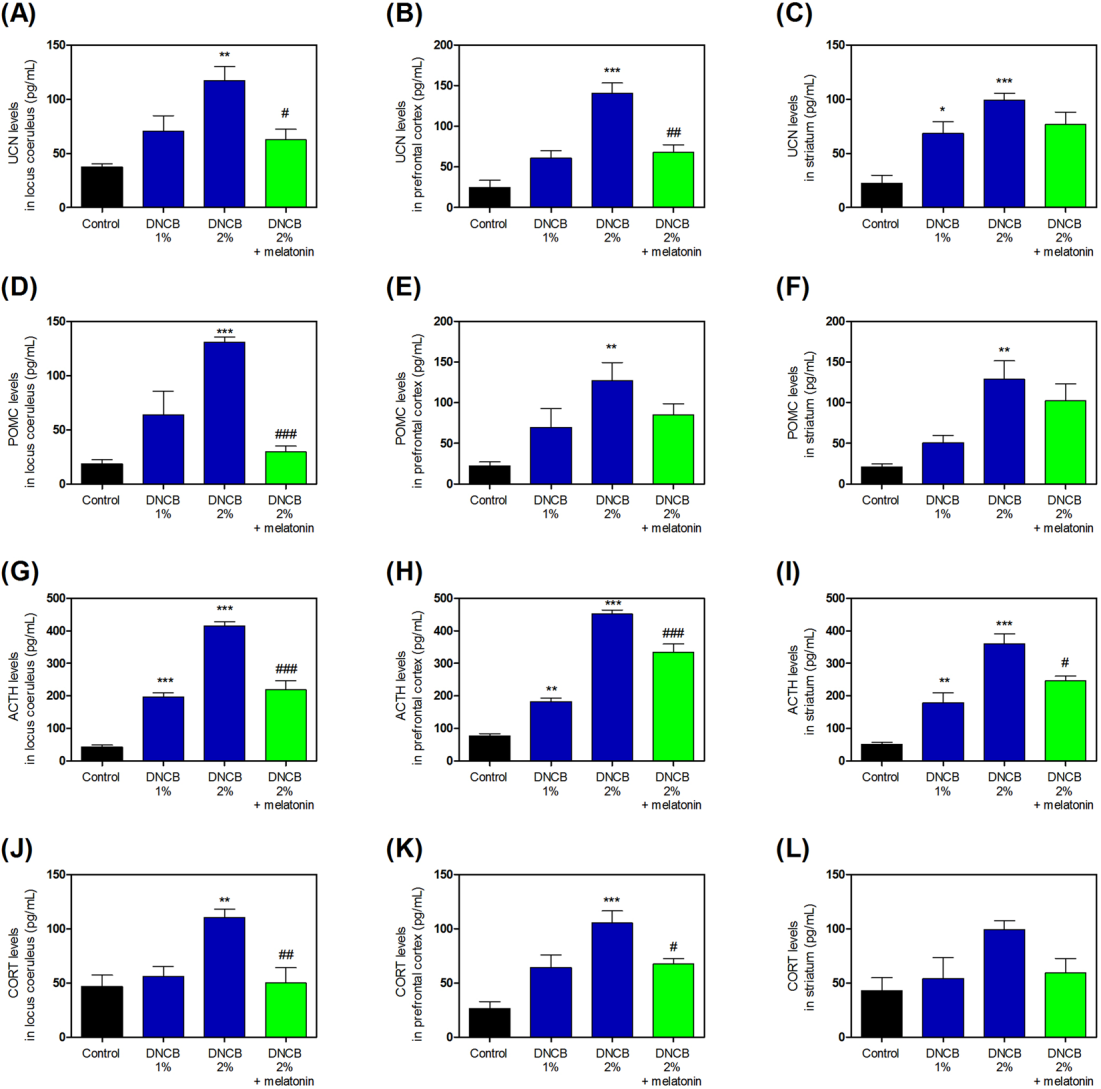
Effects of melatonin on DNCB-induced expression of the CRH-related HPA axis cascade. The levels of UCN (**A**–**C**), POMC (**D**–**F**), ACTH (**G**–**I**), and CORT (**J**–**L**) were measured by ELISA kit. Values are means ± standard error of the mean. *P < 0.05, **P < 0.01, **P < 0.01 and ***P < 0.001 compared with the control group; ^#^P < 0.05, ^##^P < 0.01, and ^###^P < 0.001 compared with the 2% DNCB-alone group. DNCB, dinitrochlorobenzene; CRH, corticotropin releasing hormone; HPA, hypothalamic-pituitary-adrenal; UCN; POMC, pro-opiomelanocortin; ACTH, adrenocorticotropic hormone; CORT, corticosterone; ELISA, enzyme-linked immunosorbent assay.

Further, to determine whether melatonin affected the stress cascade, we measured UCN, POMC, ACTH, and CORT levels. Treatment with DNCB significantly increased the levels of UCN (Fig. 5A–C, and Supplementary Table 4), while treatment with 20 mg/kg melatonin reduced the DNCB-induced UCN increase (Fig. 5A–C, and Supplementary Table 4). Treatment with DNCB significantly increased the levels of POMC (Fig. 5D–F, and Supplementary Table 4), while treatment with 20 mg/kg melatonin reduced this DNCB-induced POMC increase (Fig. 5D–F, and Supplementary Table 4). Treatment with DNCB significantly increased the levels of ACTH (Fig. 5G–I, and Supplementary Table 4), while treatment with 20 mg/kg melatonin reduced this DNCB-induced ACTH increase (Fig. 5G–I, and Supplementary Table 4). Treatment with DNCB significantly increased the levels of CORT (Fig. 5J–L, and Supplementary Table 4), while treatment with 20 mg/kg melatonin reduced this DNCB-induced CORT increase (Fig. 5J–L, and Supplementary Table 4).

### Effects of melatonin on DNCB-induced stress response cAMP-pCREB in the LC

**Figure 6:**
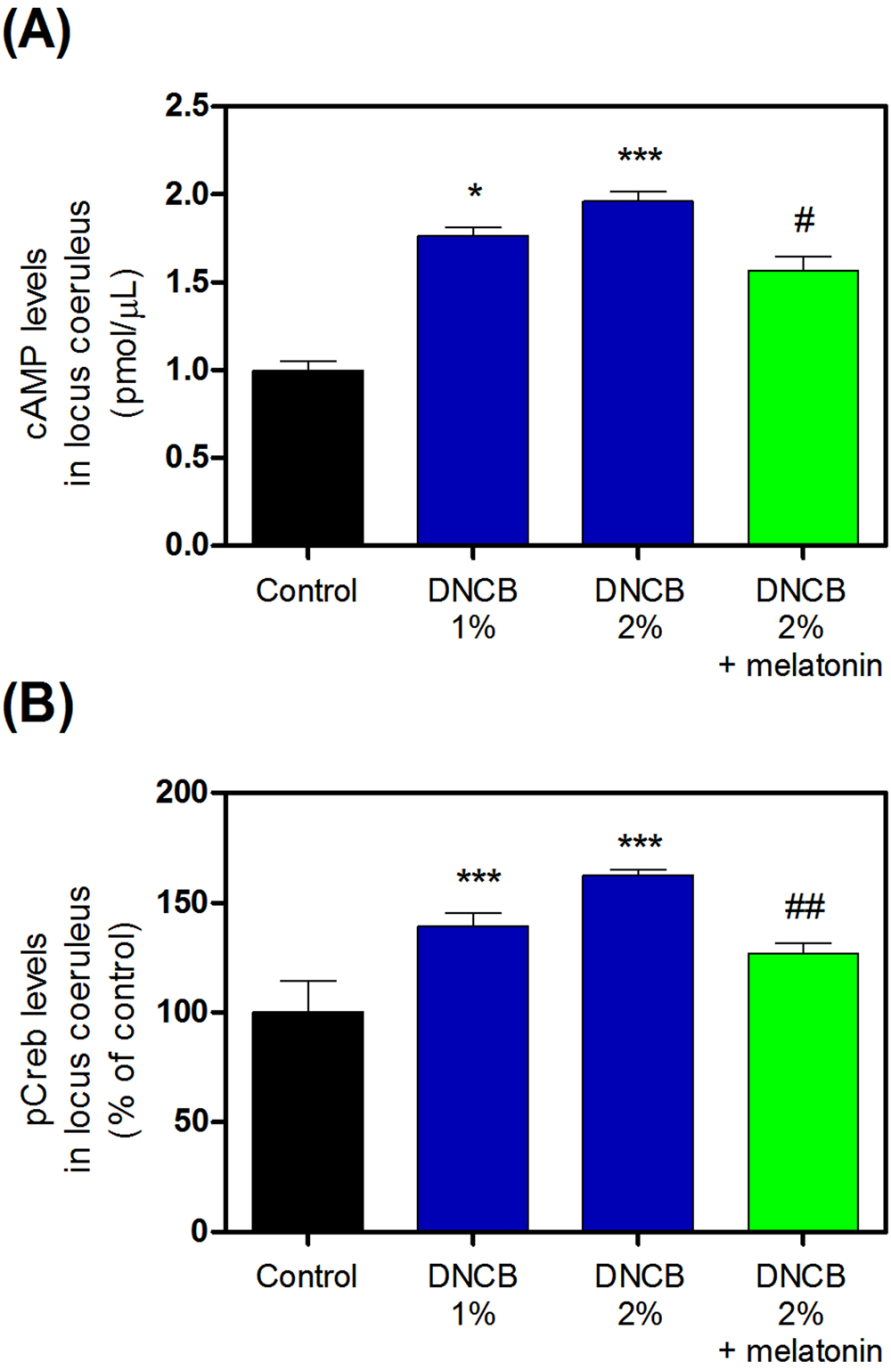
Effects of melatonin on DNCB-induced expression of the stress response cAMP-pCREB signaling in the locus coeruleus. The expression of cAMP (**A**) and pCREB (**B**) were measured by ELISA kit or immunofluorescence. Values are means ± standard error of the mean. *P < 0.05 and ***P < 0.001 compared with the control group. DNCB, dinitrochlorobenzene; cAMP, cyclic adenosine monophosphate; pCREB, phosphorylated C-element response binding protein; ELISA, enzyme-linked immunosorbent assay.

We assessed cAMP-pCREB pathway activity in the LC. Exposure of skin to 1–2% DNCB significantly increased cAMP (Fig. 6A and Supplementary Table 5) and pCREB (Fig. 6B and Supplementary Table 5) levels in the LC (Fig. 6B,C, and Supplementary Table 5). However, treatment with 20 mg/kg melatonin reduced this DNCB-induced cAMP increase (Fig. 6A and Supplementary Table 5) and pCREB (Fig. 6B and Supplementary Table 5).

### Effects of melatonin on DNCB-induced dopamine metabolic enzyme levels

**Figure 7:**
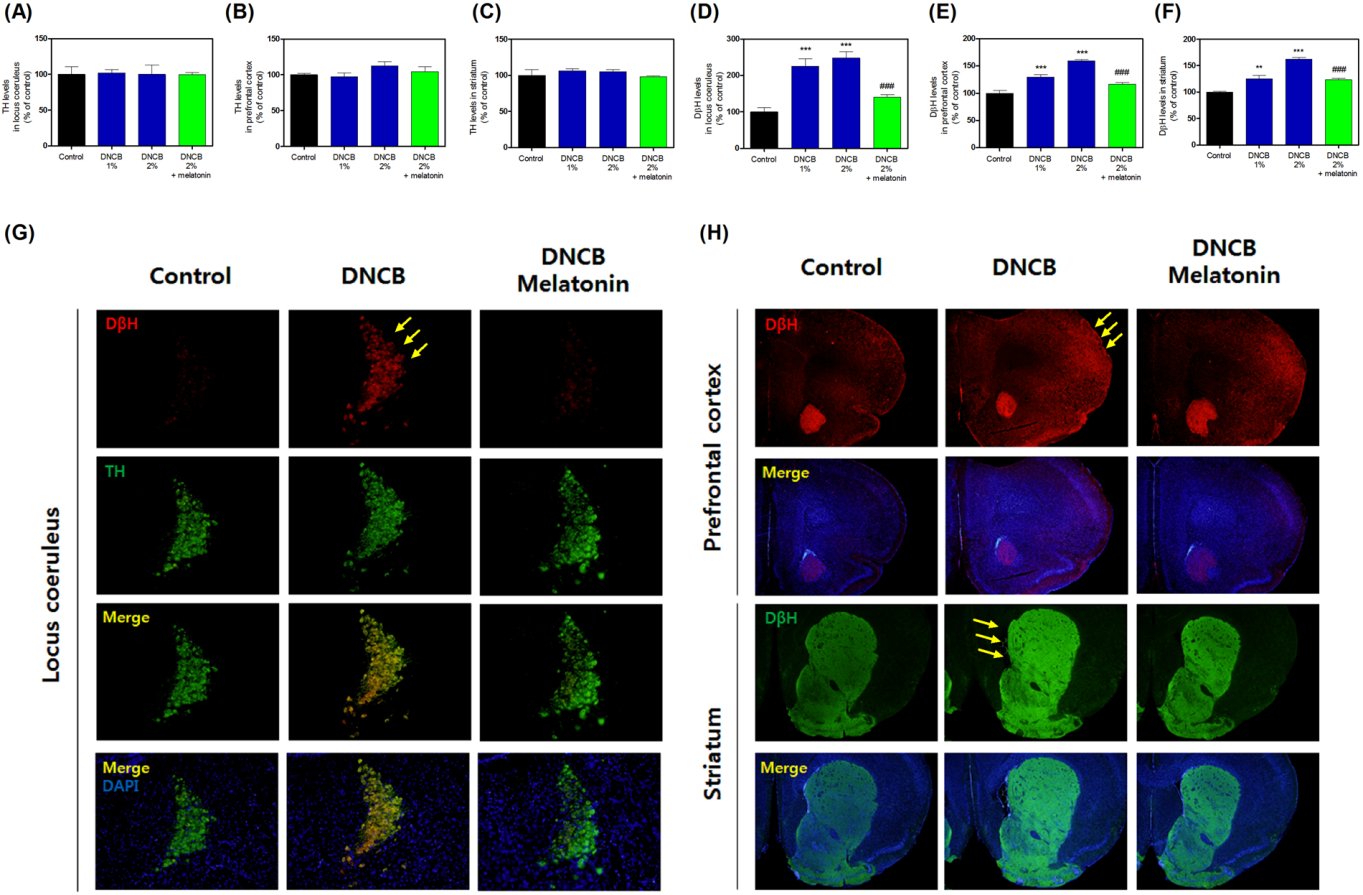
Effects of melatonin on DNCB-induced expression of the dopamine-related proteins in the locus coeruleus, prefrontal cortex, and striatum. The expression of tyrosine hydroxylase (**A**–**C**) and dopamine beta-hydroxylase (DβH; **D**–**F**) were measured by immunofluorescence. Representative photomicrographs are shown (**G**). Values are means ± standard error of the mean. *P < 0.05 and ***P < 0.001 compared with the control group.

To determine whether melatonin affects dopamine responses, we measured TH and DβH levels. Treatment with DNCB and DNCB + melatonin did not change the levels of TH compared with the control group (Fig. 7A–C, and Supplementary Table 6). However, treatment with DNCB significantly increased the levels of DβH (Fig. 7E–G, and Supplementary Table 6), while treatment with 20 mg/kg melatonin reduced the DNCB-induced DβH increase (Fig. 7D–F, and Supplementary Table 6).

### Effects of melatonin on DNCB-induced dopamine and norepinephrine levels

**Figure 8:**
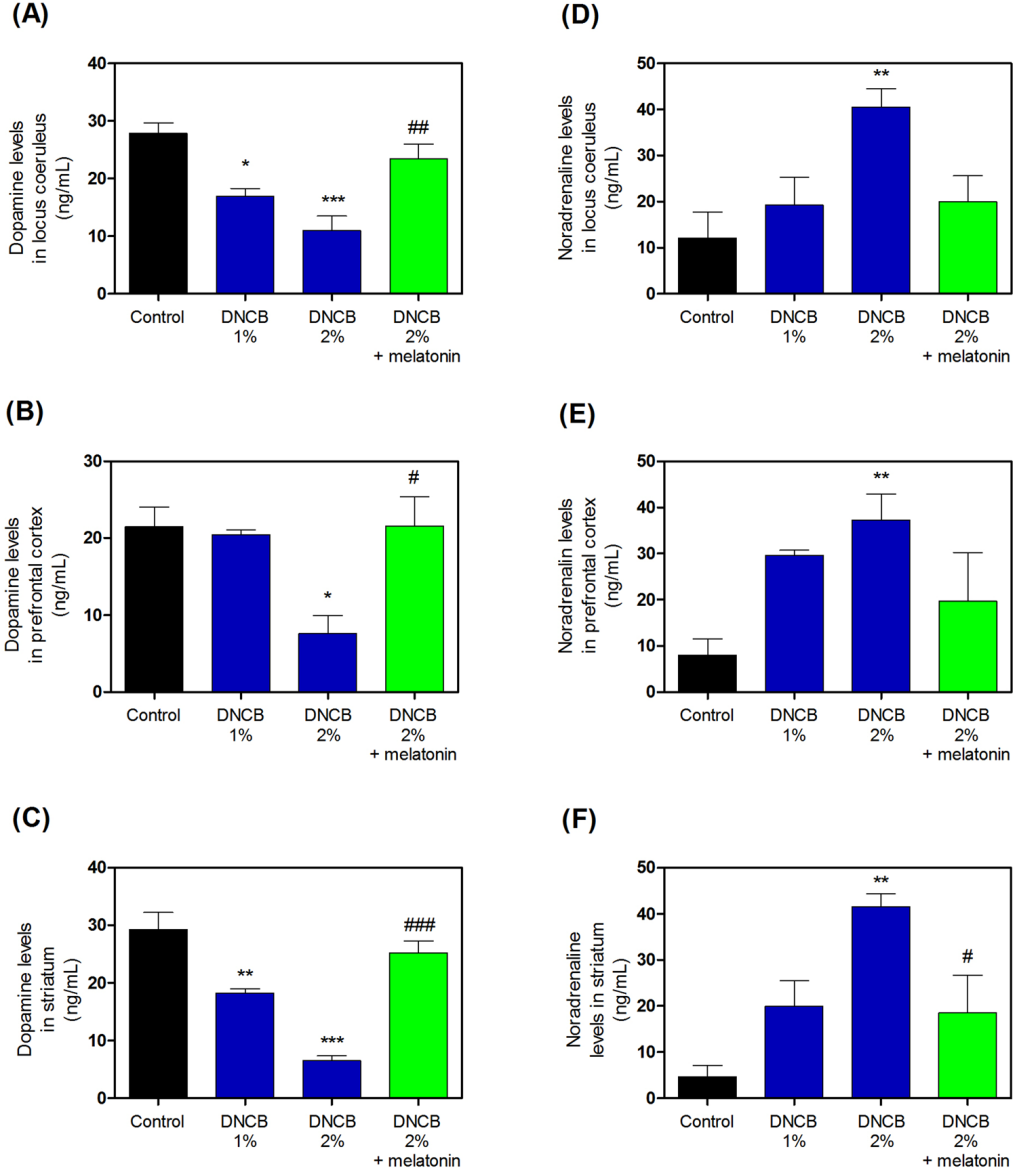
Effects of melatonin on DNCB-induced expression of the dopamine and noradrenaline contents in the locus coeruleus, prefrontal cortex, and striatum of the brain. The expression of dopamine (**A**–**C**) and noradrenaline (**D**–**F**) were measured using ELISA kits. Values are means ± standard error of the mean. *P < 0.05 and ***P < 0.001 compared with the control group. DNCB, dinitrochlorobenzene; ELISA, enzyme-linked immunosorbent assay.

We measured dopamine and norepinephrine levels after treatment with DNCB. Treatment with DNCB significantly increased the levels of dopamine (Fig. 8A–C, and Supplementary Table 7), while treatment with 20 mg/kg melatonin reduced this DNCB-induced dopamine increase (Fig. 8A–C, and Supplementary Table 7). Furthermore, treatment with DNCB significantly increased the levels of norepinephrine (Fig. 8D–F, and Supplementary Table 7), while treatment with 20 mg/kg melatonin reduced this DNCB-induced norepinephrine increase (Fig. 8D–F, and Supplementary Table 7).

### Effects of melatonin on CORT-induced dopamine and norepinephrine levels

**Figure 9:**
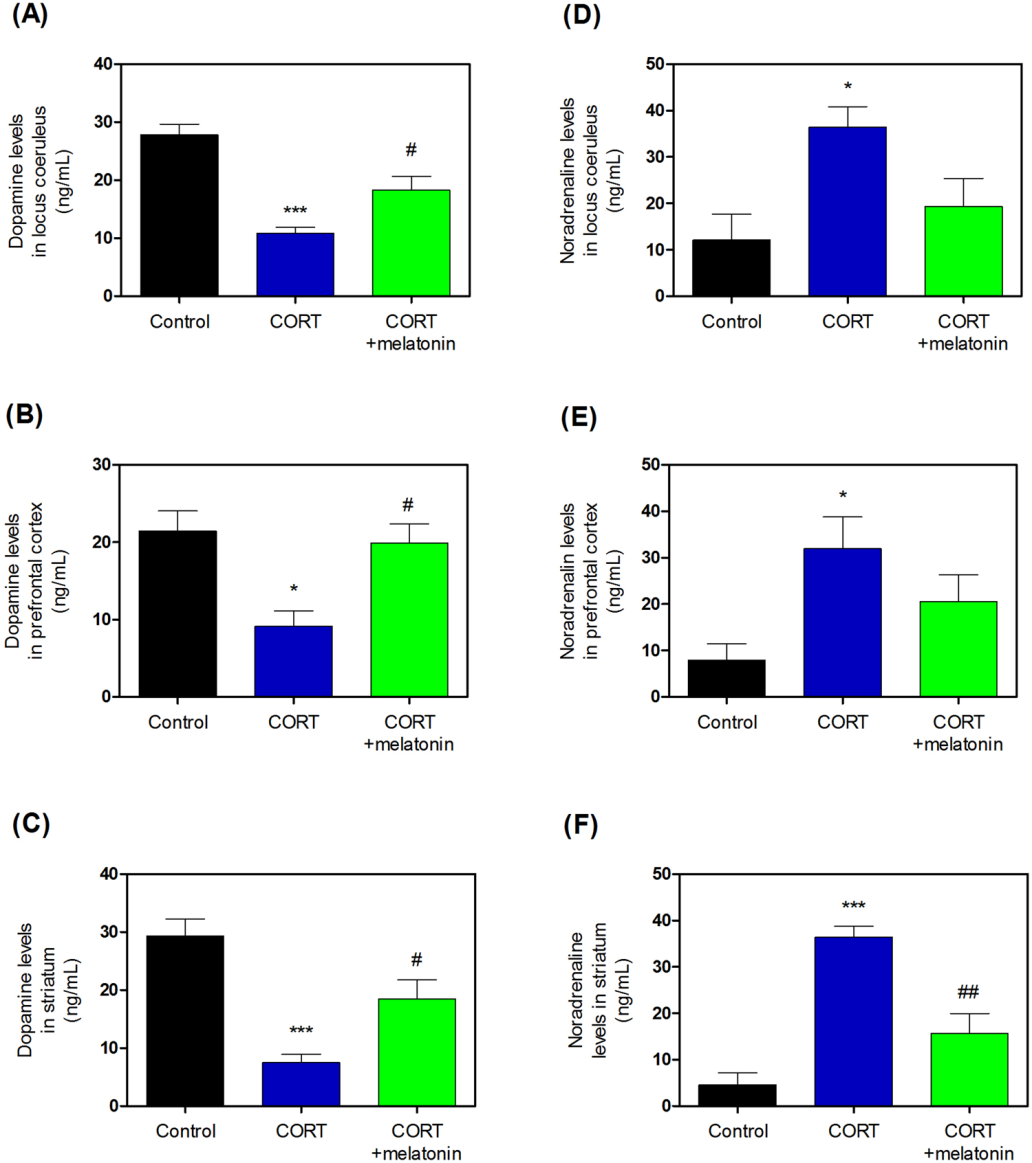
Effects of melatonin on CORT-induced expression of the dopamine and noradrenaline contents in the locus coeruleus, prefrontal cortex, and striatum of the brain. The expression of dopamine (**A**–**C**) and noradrenaline (**D**–**F**) were measured by ELISA kits. Values are means ± standard error of the mean. *P < 0.05 and ***P < 0.001 compared with the control group. CORT, corticosterone; ELISA, enzyme-linked immunosorbent assay.

We determined dopamine and norepinephrine levels after treatment with CORT. Treatment with CORT significantly increased the levels of dopamine (Fig. 9A–C, and Supplementary Table 8), while treatment with 20 mg/kg melatonin reduced this CORT-induced dopamine increase (Fig. 9A–C, and Supplementary Table 8). Furthermore, treatment with CORT also significantly increased the levels of norepinephrine (Fig. 9D–F, and Supplementary Table 8), while treatment with 20 mg/kg melatonin reduced this CORT-induced norepinephrine increase (Fig. 9D–F, and Supplementary Table 8).

## Discussion

The data presented herein demonstrate that the atopy-induced stress response significantly increased the presence of signaling molecules involved in ADHD. This response is due to factors such as dopamine and noradrenalin imbalances (via the up-regulation of DβH), which exacerbate HPA dysfunction and suppress the melatonin feedback system. Atopic stress led to: (1) induction of CRH-related and suppressing melatonin signaling in the LC and ST; (2) increased levels of DβH that did not alter TH levels; and (3) decreased dopamine levels and increased noradrenalin levels. Melatonin reversed these effects on dopamine and noradrenalin via stimulation of the normal HPA axis. To the best of our knowledge, in the first study to demonstrate that AD-caused psychologic stress increases catecholamine dysfunction and accelerated the development of psychiatric comorbidities, such as ADHD via dysregulation of the HPA/melatonin signaling pathways.

ADHD, characterized by inattention, hyperactivity/impulsivity, or both, is one of the most common psychiatric disorders of childhood^43^. Approximately one third of medication-free children with ADHD experience chronic sleep-onset insomnia^43–45^. Although the safety and efficacy of melatonin treatment for sleep-onset insomnia in children without ADHD have been well-documented, melatonin efficacy has not been studied in medication-free children with ADHD and sleep-onset insomnia; this patient group is of special interest for several reasons^46,47^. First, medication-free children with both disorders exhibit a delayed evening increase in endogenous melatonin levels, and this phase delay predicts strengthening of the sleep phase, which normalizes the effect of exogenous melatonin in children without ADHD^46^. Second, because treating sleep-related disorders other than insomnia improves daytime function in children with ADHD, treating insomnia may have important consequences for ADHD treatment strategies^48–50^. The night time circadian rise in melatonin levels correlates with a nighttime circadian drop in glucocorticoids^51,52^. Any chronic, late-night stressor (e.g., shift-work^53^) can result in excessively high nighttime cortisol levels, which may impair the normal morning circadian increase in corticosteroid levels^54^. It is known that circadian rhythms are highly related to the HPA axis, which is a key neuroendocrine mediator of physiological responses to psychological stressors^55–57^. In a previous study, we found that hyper-activation of HPA axis induced by atopic chronic psychological stress was associated with neurotoxicity and cognitive impairment, and it also blunted neuroendocrine responses to stress^25^. In this and our previous paper, we focused on the idea that neuroendocrine contribution to the responses in skin to stress is promoted, in part, by local synthesis of all elements of the HPA axis. Skin has the ability to synthesize glucocorticoids from cholesterol or steroid intermediates of systemic origin^58,59^. By interacting with glucocorticoid receptors, they regulate skin immune functions as well as functions and phenotype of the epidermal, dermal and adnexal compartments^58,59^. Most of the biochemical (enzyme and transporter activities) and regulatory principles of cutaneous glucocorticosteroidogenesis (neuropeptides mediated activation of cAMP and protein kinase A dependent pathways) are similar to those operating in classical steroidogenic organs^58–61^.

According to a recent report, stimulation of cutaneous corticosteroidogenesis can occur via this skin homologue of the HPA axis which is dependent on the functional activating CRHR and processing POMC^60,61^. Specifically, the stimulating cortisol synthesis by IL1 is intriguing because IL1 serves as a local signal of skin inflammatory injury^60,61^. It is possible that IL1 may stimulate corticosteroidogenesis indirectly, through up-regulating CRH or POMC peptides or by itself, which has been reported to occur in the adrenals^60,61^. CRH, in addition to indirect stimulation may directly stimulate local corticosteroidogenesis because it increases cAMP^62,63^. A similar direct action of CRH on adrenocortical region has been reported^62,63^. An additional regulating-mechanism involves the activation or inactivation of glucocorticosteroids by locally expressed cortisone reductase, such as 11β-hydroxysteroid dehydrogenase type1 and 2^62–64^.

In this and our previous paper, we confirmed that melatonin control anti-inflammation by regulating the HPA axis via the skin. Melatonin plays an important role in the regulating circadian timing and neuro-immunologic function^54,65^. Recent studies have linked decreases in melatonin output to insomnia in aged patients and sleep disturbances in patients with Alzheimer’s disease^25,66–69^. In AD, a severity score ≥48.7 predicts elevated levels of immunoglobulin, sensitive allergen, and poor sleep efficiency, possibly because of reduced nocturnal melatonin secretion, pruritus, and associated scratching^25,69^. One study reported disrupted melatonin secretion in eczema, possibly due to partial action of the sympathetic nervous reduction that regulates secretion of melatonin^17,25^. Recent studies have shown that melatonin is synthesized in numerous extrapineal sites^54,65,70^. Additionally, melatonin is regulated by rapid metabolism in the liver and peripheral organs including the skin^54^. Several researchers have proposed that melatonin and its metabolites affect skin functions and structures through actions mediated by intracutaneously expressed cell-surface and putative nuclear receptors^54,70^. Melatonin exerts both receptor-dependent and receptor-independent protective effects against oxidative stress and can attenuate environmental skin stressor–induced damage^71^. The effects of the common environmental skin stressors are modulated by melatonin via a complex intra-cutaneous melatonergic anti-oxidative system, with ultraviolet radiation-enhanced melatonin metabolism generating bioactive melatonin metabolites such as acetyl-N-formyl-5-methoxykynurenamine^70,71^. These properties suggest that melatonin is an important endogenous effector of intracutaneous stress responses. In fact, there is a shortage of information concerning the regulation of HPA axis-melatonin-circadian rhythms on the glucocorticosteroidogenesis signaling system in skin^61–63^. However, previously, we found that NC/Nga mice exposed to atopic stress exhibited substantial reductions in hypothalamic melatonin membrane receptor expression and in hypothalamic and intracutaneous melatonin expression^25^. Additionally, historically, oriental medicine has suggested a similar connection with the thought that biological rhythm is an inherent connotation of “harmony between human and nature”^72^. Our previous study did not show whether intracutaneous melatonin affects the brain, but many other studies have suggested that it has an important effect. Further research is certainly needed, but our observation of decreased melatonin levels in the LC, PC, and ST in DNCB-exposed NC/Nga mice supports this hypothesis.

In the present study, we tried to investigate effects of stress in the LC. Some anti-depressants as well as the ADHD medication atomoxetine, are believed to act on LC neurons^73,74^. The LC is responsible for mediating several sympathetic effects of stress; it is activated by stress and responds by increasing norepinephrine secretion, which in turn alters cognitive function (through the PC), increases motivation (through nucleus accumbens), and activates the HPA axis^75^. After HPA axis stimulation, norepinephrine stimulates the secretion of CRH from the hypothalamus, which induces ACTH release from the anterior pituitary and subsequent cortisol synthesis in the adrenal glands^55,76,77^. Norepinephrine released from the LC will inhibit its own production, and CRH will inhibit its own production while causing the LC to increase norepinephrine production^73^. Thus, to determine whether mechanisms of the dopamine and noradrenaline imbalance caused by atopic stress were regulated by melatonin, we measured ADHD signaling patterns in an NC/Nga atopic mouse model. We showed that DNCB significantly increased levels of HPA axis-related response substances such as CRH, CRHR, UCN, POMC, ACTH, and CORT, while treatment with melatonin significantly reduced these levels in DNCB-treated mice. Further, corticosteroid-mediated psychological stress responses utilize various signal transduction systems^78–80^. Studies have shown that in hippocampal neurons or slices, ERK1/2 respond to stressful stimuli through the transcription factor CREB (cyclic response element binding protein), which activates c-Fos via CRE sites in promoter regions^79^. Additionally, stress increases phosphorylating CREB^80^. Moreover, the PI3K-cAMP-CREB pathway activity is elevated in the noradrenergic neurons of the LC, which is the main source of noradrenaline in the brain^37^. PI3Kc gene KO mice show increased CREB activation via elevation of cAMP levels in the LC and alters the dopamine/noradrenaline balance in the PC and ST^7,37^. These changes facilitate the development of core ADHD-related phenotypes, including hyperactivity and attention deficit, as well as secondary features such as memory and social impairments^7,37^. Overexpression of CREB in the LC of normal animals produces similar behavioral changes, and down-regulation of CREB activity in the LC of mutant mice reverses the phenotype^7,37^. In the present study, we observed significantly increased cAMP and pCREB expression in the LC; subchronic melatonin reduced cAMP and pCREB expression in DNCB-treated mice.

Further, we examined DβH and TH levels. DβH is anenzyme that converts DA into NE and is coreleased with catecholamines^81^. TH is involved in the conversion of phenylalanine to dopamine^81^. As the rate-limiting enzyme in the synthesis of catecholamines, TH plays a key role in the physiology of adrenergic neurons; TH is regularly used as a marker for dopaminergic neurons^81–83^. DβH is a genetic marker and may reflect individual susceptibility to developing psychosis in the context of exposure to traumatic events^84^. Generally, adrenal catecholamines are known to mediate many of the physiological consequences of the “fight or flight” response to stress^84,85^. However, the mechanisms by which the long-term responses to repeated stress exposure are mediated are not well understood. According to McMahon *et al*., both TH and DβH levels are elevated by single and repeated exposure to immobilization stress^84,85^. Surprisingly, we observed no or slight change in TH expression in the LC. However, DβH levels were significantly upregulated in the LC, PC, and ST of DNCB-treated mice. Further, subchronic melatonin reduced DβH expression. We also observed significantly decreased dopamine and increased noradrenaline levels in the LC, PC, and ST of DNCB-treated mice; subchronic melatonin treatment reduced these levels. This pattern demonstrated similar results in CORT injection model. In this study, we suggested that the imbalance resulting from increased release of dopamine and noradrenaline for potential modulation of physiological or immunological responses is the consequence of the upregulated DβH expression and the unchanged TH expression in DNCB exposed to atopic stress. However, future research will be needed to analyze the specific mechanisms system. Thus, AD-related neuropsychological stress caused the relationship of normal glucocorticoid/melatonin disruption and accelerated dopamine dysregulation.

This study has some limitations which have to be pointed out. We did not assess whether atopic stress induced ADHD behavioral pattern of chronic stress, because the scratching behavior and memory impairments interfered with the rodent behavioral tasks. Several pilot experiments were conducted, but they were unsuccessful. Additionally, melatonin is correlated with the circadian rhythm phase shift of the circadian clock; hence, a change in sleep-wake time is expected. However, the experiments were unsuccessful as itching interfered with the sleep-wake time and the sleep-wake time criterion was unclear. Thus, future studies will need to analyze the wake-sleep states, specific response of stress, and their potential behavioral outcomes.

## Conclusions

**Figure 10:**
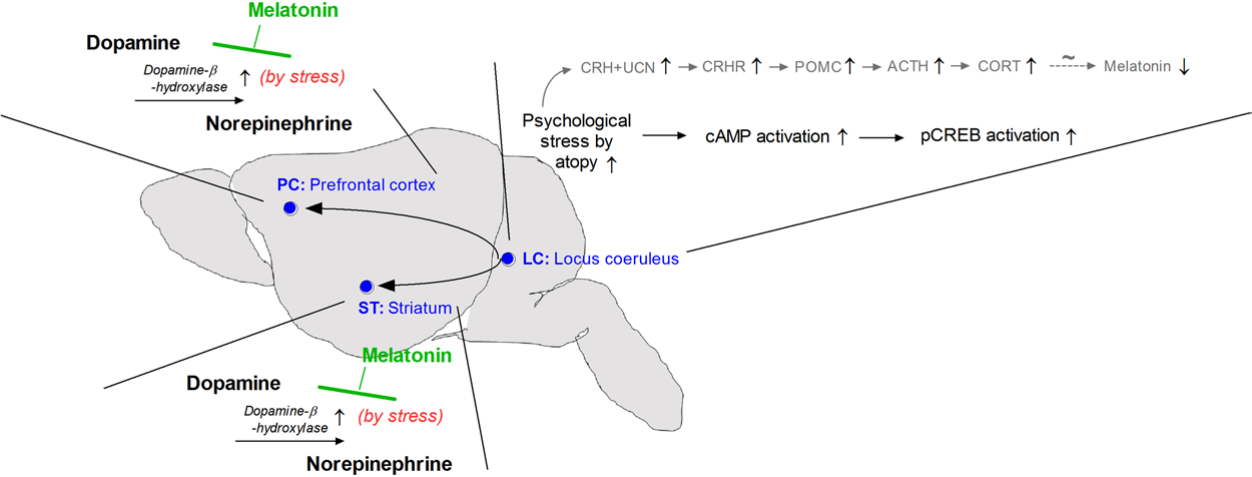
Schematic of the mechanism proposed for the effects of melatonin on the hypothalamic-pituitary-adrenal (HPA) axis and attention deficit hyperactivity disorder (ADHD) pathogenesis.

In conclusion, we found that atopic stress accelerated HPA-axis dysfunction, increased norepinephrine, and decreased dopamine (Fig. 10). Atopic stress induced HPA axis-related response dysfunction and ERK-CREB signaling pathway, which reduced melatonin levels in the LC, PC, and ST. Moreover, atopy-induced stress accelerated DβH level increases and dopamine consumption. Further, we discovered that melatonin inhibited reduced dopamine consumption by the inhibition of DβH via regulating HPA in AD models. Therefore, our findings suggest that the CORT-melatonin disequilibrium might contribute to dysfunction of dopamine by causing or enhancing neurodegeneration, which could lead to disorders such as ADHD.

## Electronic supplementary material

Supplementary table 1–8
