# Supplementary material for: Melatonin inhibits attention-deficit/hyperactivity disorder caused by atopic dermatitis-induced psychological stress in an NC/Nga atopic-like mouse model
Source: Sci Rep. 2018 Oct 8;8:14981. doi: 10.1038/s41598-018-33317-x (PMC6175954; doi:10.1038/s41598-018-33317-x)
Supplement: Supplementary file 1 — Supplementary table 1–8 [file 41598_2018_33317_MOESM1_ESM.docx]

**Supplemental information – *Table***

**Melatonin inhibits attention-deficit/hyperactivity disorder caused by atopic dermatitis-induced psychological stress in an NC/Nga atopic-like mouse model**

***Running title: Melatonin inhibits dopamine dysregulation by atopy.***

**Gunhyuk Park^1, 2*†^, Young-Suk Jung^3^, Moon-Ki Park^4^, Chae Ha Yang^5^, Yong-ung Kim^4*^**

*^1^The K-herb Research Center, Korea Institute of Oriental Medicine, Daejeon, Republic of Korea*

*^2^Ektos industries Co., Lid., Daejeon, Republic of Korea*

*^3^College of Pharmacy, Pusan National University, Busan, Republic of Korea*

*^4^Department of Pharmaceutical Engineering, College of Biomedical Science, Daegu Haany University, Gyeongsan , Republic of Korea*

*^5^Department of Physiology, College of Korean Medicine, Daegu Haany University, Daegu, Republic of Korea*

***Co-corresponding-author 1**: Gunhyuk Park, Ph.D.

*^1^*Ektos Industries Co., Lid., Daejeon, Republic of Korea

*^2^*Department Institute of Human-Environment Interface Biology, Medical Research Center, Seoul National University College of Medicine, Seoul, Republic of Korea; Department of Dermatology, Seoul National University College of Medicine, Seoul, Republic of Korea

E-mail address: parkgunhyuk@gmail.com and gpark@snu.ac.kr

***Co-corresponding-author 2**: Yong-ung Kim, Ph.D.

Department of Pharmaceutical Engineering, College of Biomedical Science, Daegu Haany University, Gyeongsan, Republic of Korea

E-mail address: ykim@dhu.ac.kr

*^†^* **Present address**: Gunhyuk Park, Ph.D., Senior researcher

Department Institute of Human-Environment Interface Biology, Medical Research Center, Seoul National University College of Medicine, Seoul, Republic of Korea; Department of Dermatology, Seoul National University College of Medicine, Seoul, Republic of Korea

**Table 1.** Effects of CORT on expression of DβH in SH-SY5Y cells.

| **DβH levels** | **CORT (µM)** | | |  |
| --- | --- | --- | --- | --- |
| **(% of control)** | **—** | **5** | **10** | **20** |
|  | 100.00±1.29 | 91.49±2.08 | 80.19±1.97 | 57.01±4.23 |

| **DβH levels** | **Melatonin (µM)** | | |  |
| --- | --- | --- | --- | --- |
| **(% of control)** | **—** | **5** | **10** | **20** |
|  | 100.00±2.54 | 104.01±2.07 | 123.90±5.26 | 139.20±6.01 |

**Table 2.** Effects of DNCB on expression of melatonin in brain.

| **Melatonin levels** | **DNCB** | | |
| --- | --- | --- | --- |
| **(pg/mL)** | **—** | **1%** | **2%** |
| **LC** | 39.60±5.57 | 28.23±9.32 | 17.72±2.09 |
| **PC** | 42.53±4.34 | 26.80±4.59 | 16.65±10.48 |
| **ST** | 47.37±4.04 | 29.71±5.66 | 27.86±7.02 |

**Table 3.** Effects of melatonin on DNCB-induced expression of CRH and CRHR in locus coeruleus, prefrontal cortex, and striatum of brain.

| **CRH levels** | **DNCB** | | | |
| --- | --- | --- | --- | --- |
| **(pg/mL)** | **—** | **1%** | **2%** | **2%+melatonin** |
| **LC** | 28.73±4.23 | 70.30±9.19 | 99.25±17.99 | 77.33±20.43 |
| **PC** | 35.75±15.56 | 84.53±24.58 | 146.03±9.67 | 64.85±7.36 |
| **ST** | 31.30±11.15 | 99.10±15.29 | 105.68±28.78 | 52.88±8.48 |

| **CRH-R levels** | **DNCB** | | | |
| --- | --- | --- | --- | --- |
| **(pg/mL)** | — | **1%** | **2%** | **2%+melatonin** |
| **LC** | 6.85±0.67 | 8.28±0.53 | 10.65±0.48 | 8.87±0.18 |
| **PC** | 6.60±0.80 | 8.84±0.15 | 10.67±0.53 | 7.27±0.76 |
| **ST** | 7.80±0.72 | 9.50±0.12 | 11.03±0.29 | 8.65±0.59 |

**Table 4.** Effects of melatonin on DNCB-induced expression of the CRH-related HPA axis cascade.

| **UCN levels** | **DNCB** | | | |
| --- | --- | --- | --- | --- |
| **(pg/mL)** | **—** | **1%** | **2%** | **2%+melatonin** |
| **LC** | 37.72±2.50 | 70.16±14.84 | 117.27±12.77 | 62.76±9.47 |
| **PC** | 24.59±9.19 | 60.80±9.22 | 140.73±12.49 | 67.89±9.46 |
| **ST** | 22.45±7.39 | 68.29±10.98 | 99.21±6.27 | 76.78±11.43 |

| **POMC levels** | **DNCB** | | | |
| --- | --- | --- | --- | --- |
| **(pg/mL)** | **—** | **1%** | **2%** | **2%+melatonin** |
| **LC** | 18.40±4.10 | 63.83±21.98 | 130.80±4.97 | 29.68±5.28 |
| **PC** | 22.15±5.21 | 69.93±22.86 | 127.25±21.84 | 85.43±13.36 |
| **ST** | 21.25±3.75 | 50.95±8.53 | 128.93±22.64 | 102.75±20.47 |

| **ACTH levels** | **DNCB** | | | |
| --- | --- | --- | --- | --- |
| **(pg/mL)** | **—** | **1%** | **2%** | **2%+melatonin** |
| **LC** | 43.62±5.52 | 197.30±12.69 | 415.36±13.33 | 219.08±28.02 |
| **PC** | 76.84±7.22 | 183.28±10.76 | 451.77±12.78 | 335.21±24.60 |
| **ST** | 50.93±7.07 | 179.08±30.58 | 360.48±30.65 | 245.89±14.76 |

| **CORT levels** | **DNCB** | | | |
| --- | --- | --- | --- | --- |
| **(pg/mL)** | **—** | **1%** | **2%** | **2%+melatonin** |
| **LC** | 46.82±10.34 | 55.86±9.46 | 110.43±7.94 | 50.07±13.89 |
| **PC** | 26.57±6.27 | 64.14±11.81 | 105.46±10.97 | 67.39±5.03 |
| **ST** | 42.64±12.18 | 54.14±19.32 | 99.25±8.09 | 59.39±13.41 |

**Table 5.** Effects of melatonin on DNCB-induced expression of the stress response cAMP-pCREB signaling in the locus coeruleus.

| **cAMP levels** | **DNCB** | | | |
| --- | --- | --- | --- | --- |
| **(pmol/µL)** | **—** | **1%** | **2%** | **2%+melatonin** |
|  | 1.00±0.06 | 1.76±0.05 | 1.96±0.05 | 1.56±0.08 |

| **pCreb levels** | **DNCB** | | | |
| --- | --- | --- | --- | --- |
| **(% of control)** | **—** | **1%** | **2%** | **2%+melatonin** |
|  | 100.00±14.20 | 139.61±5.84 | 162.45±2.74 | 126.96±4.67 |

**Table 6.** Effects of melatonin on DNCB-induced expression of the dopamine-related proteins in the locus coeruleus, prefrontal cortex, and striatum.

| **TH levels** | **DNCB** | | | |
| --- | --- | --- | --- | --- |
| **(% of control)** | **—** | **1%** | **2%** | **2%+melatonin** |
| **LC** | 100.00±10.53 | 102.25±4.21 | 100.26±13.02 | 99.25±3.37 |
| **PC** | 100.00±1.95 | 97.50±4.76 | 112.50±5.81 | 104.58±6.47 |
| **ST** | 100.00±7.65 | 106.47±2.42 | 105.22±2.53 | 97.91±1.54 |

| **DβH levels** | **DNCB** | | | |
| --- | --- | --- | --- | --- |
| **(% of control)** | **—** | **1%** | **2%** | **2%+melatonin** |
| **LC** | 100.00±12.67 | 224.75±10.85 | 247.52±17.02 | 140.59±6.95 |
| **PC** | 100.00±5.33 | 129.90±4.18 | 159.19±2.21 | 116.57±2.81 |
| **ST** | 100.00±1.60 | 125.37±6.01 | 162.37±3.31 | 123.26±3.08 |

**Table 7.** Effects of melatonin on DNCB-induced expression of the dopamine and noradrenaline contents in the locus coeruleus, prefrontal cortex, and striatum of brain.

| **Dopamine levels** | **DNCB** | | | |
| --- | --- | --- | --- | --- |
| **(ng/mL)** | **—** | **1%** | **2%** | **2%+melatonin** |
| **LC** | 27.83±1.83 | 16.94±1.34 | 10.94±2.54 | 23.47±2.49 |
| **PC** | 21.47±2.57 | 20.45±0.65 | 7.60±2.37 | 21.56±3.86 |
| **ST** | 29.29±2.98 | 18.24±0.74 | 6.53±0.81 | 25.27±2.03 |

| **Noradrenalin levels** | **DNCB** | | | |
| --- | --- | --- | --- | --- |
| **(ng/mL)** | **—** | **1%** | **2%** | **2%+melatonin** |
| **LC** | 12.11±5.62 | 19.27±6.00 | 40.58±3.88 | 19.93±5.70 |
| **PC** | 7.96±3.55 | 29.62±1.09 | 37.26±5.70 | 21.03±10.51 |
| **ST** | 4.67±2.47 | 19.98±5.48 | 41.57±2.78 | 18.51±8.08 |

**Table 8.** Effects of melatonin on CORT-induced expression of the dopamine and noradrenaline contents in the locus coeruleus, prefrontal cortex, and striatum of brain.

| **Dopamine levels** | **CORT (mg/kg)** | | |
| --- | --- | --- | --- |
| **(ng/mL)** | **—** | **20** | **20 + melatonin** |
| **LC** | 27.83±1.83 | 10.82±1.06 | 18.26±2.43 |
| **PC** | 21.47±2.57 | 9.11±2.02 | 19.90±2.43 |
| **ST** | 29.29±2.98 | 7.49±1.43 | 18.51±3.25 |

| **Noradrenalin levels** | **CORT (mg/kg)** | | |
| --- | --- | --- | --- |
| **(ng/mL)** | **—** | **20** | **20 + melatonin** |
| **LC** | 12.11±5.62 | 36.42±4.41 | 19.30±6.08 |
| **PC** | 7.96±3.55 | 31.95±6.84 | 20.56±5.73 |
| **ST** | 4.67±2.47 | 36.39±2.46 | 15.60±4.40 |
